# Supplementary material for: Evidence for ground state coherence in a two-dimensional Kondo lattice
Source: Nat Commun. 2023 Nov 2;14:7005. doi: 10.1038/s41467-023-42803-4 (PMC10622499; doi:10.1038/s41467-023-42803-4)
Supplement: Supplementary file 1 — Supplementary Information [file 41467_2023_42803_MOESM1_ESM.pdf]

Supplementary Information for

Evidence for ground state coherence in a two-dimensional Kondo lattice

Wen Wan, Rishav Harsh, Antonella Meninno, Paul Dreher, Sandra Sajan, Haojie Guo,

Ion Errea, Fernando de Juan* and Miguel M. Ugeda*

** Corresponding authors:* fernando.dejuan@dipc.org *and* mmugeda@dipc.org

This PDF file includes: **Page**

1. Large-scale morphology of 1T-$TaSe_2$/1H-$TaSe_2$ heterobilayers 2
2. Temperature-dependent low-energy electronic structure 3
3. Lower and upper Hubbard band in 1T-$TaSe_2$ on 1H-$TaSe_2$ 4
4. The 1H-$TaSe_2$/BLG substrate 5
5. Uncertainty band of Δ 6
6. Measurement of the non-linear behavior of Δ with the magnetic field 7
7. Absence of magnetism in single-layer $TaSe_2$ 8
8. Band structure calculations and estimate of Kondo hybridization 10
9. References 13

1. Large-scale morphology of 1T-$TaSe_2$/1H-$TaSe_2$ heterobilayers

The molecular beam epitaxy (MBE) growth of our 1T-$TaSe_2$/1H-$TaSe_2$ heterobilayers leads to a morphology as that shown in the Supplementary Figure 1. Large islands (~200 nm in diameter) of single-layer 1H-$TaSe_2$ grow on BLG, often decorated by second upper layers (~60 nm). Most of these upper layers belong to the 1T polytype, thus forming the 1T-$TaSe_2$/1H-$TaSe_2$ heterobilayer studied here.

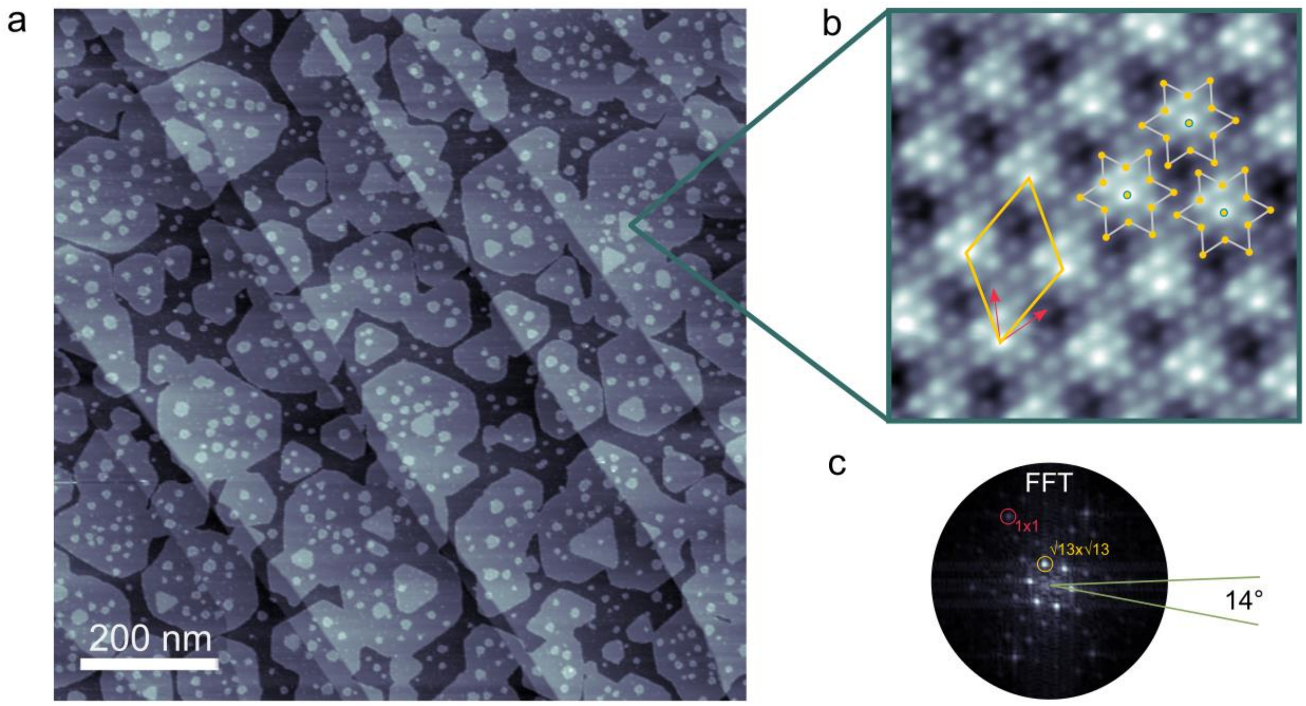

Supplementary Figure 1. Morphology of the 1T-$TaSe_2$/1H-$TaSe_2$ heterobilayer. **a**, Large-scale STM image of $TaSe_2$ on BLG/SiC(0001)) (V_s = 1.8 V, I = 0.02 nA, T = 4.2 K). **b**, Atomically resolved STM image taken on 1T-$TaSe_2$/1H-$TaSe_2$ (V_s = -0.25 V, I = 0.1 nA, T = 0.34 K). Three Star-of- David clusters are overlaid. The yellow rhombus shows the CDW supercell and the red arrows indicate the directions of the atomic registry. **c**, Fast Fourier transform (FFT) of the STM image in **b**, which shows the ~14º rotational misalignment between the CDW and the atomic lattice.

2. Temperature-dependent low-energy electronic structure

Low-bias STS measurements at 4.2 K display a single peak centered around E_F, which turns into a double-peak feature at T = 0.34 K. We have probed the evolution of the low-energy electronic structure in the range 0.34 K – 4.5 K. Supplementary Figure 2a shows a series of dI/dV spectra taken on a Star-of-David that showing the two-peak feature at 0.34 K. The position of the peaks is temperature independent and the only visible effect is the gradual filling of the conductance at E_F, which is attributed to thermal broadening. Δ remains nearly constant with T (Supplementary Fig. 2b) and cannot be further identified beyond 4-5 K due to thermal broadening. In conclusion, these results show that the emergence of two peaks as T lowers is not gradual (T-dependent process) but rather due to an increase of STS resolution. It is therefore plausible that the emergence of coherence of the magnetic moments occur at higher temperatures.

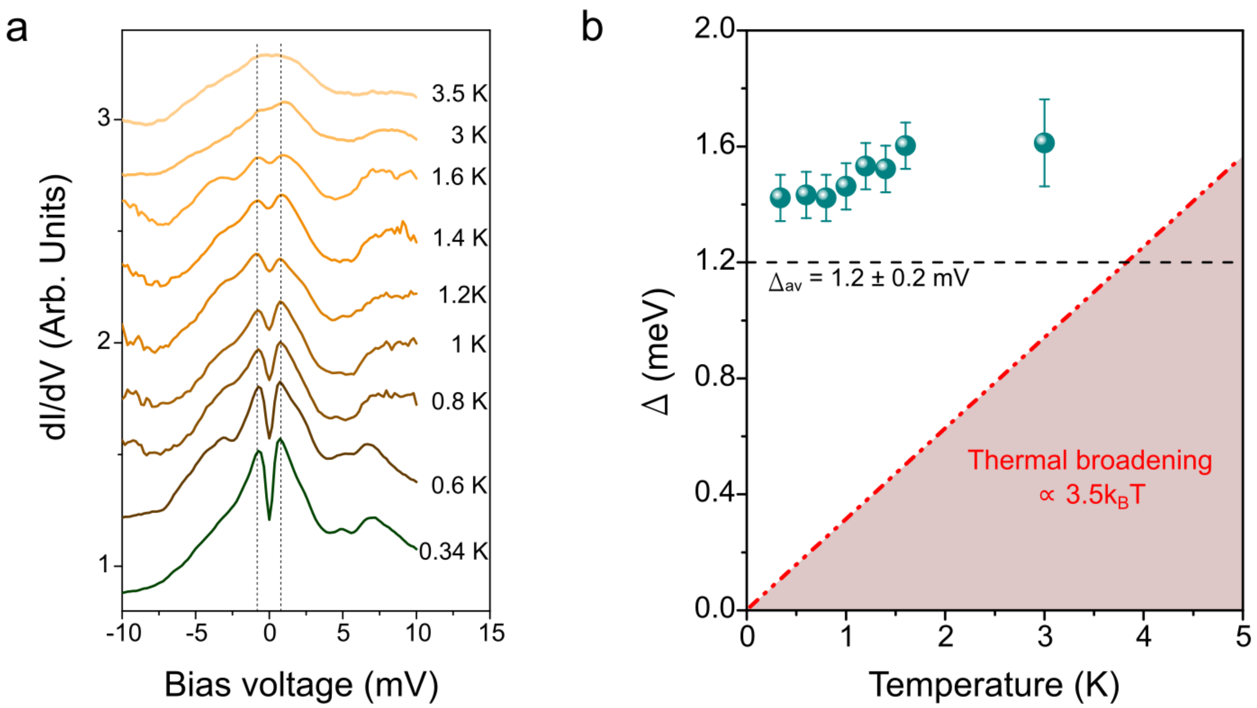

Supplementary Figure 2. Temperature dependence of the low-energy electronic structure. **a**, Series of low-bias dI/dV spectra taken in the 0.34 – 3.5 K range ($V_{a.c.}$ = 30 μV). **b**, Δ vs. T for the dI/dV set in **a**. The shaded area indicates the region where Δ cannot be resolved via STS due to thermal broadening.

3. Lower and upper Hubbard band in 1T-$TaSe_2$ on 1H-$TaSe_2$

The realization of the Kondo effect (either for a single impurity or for a Kondo lattice) implies the existence of two localized resonances in tunneling above and below the Fermi level, which correspond to the localized level with energy ϵ_f occupied by a single electron, and the doubly occupied level with an energy $\epsilon_f + U$ due to the local Coulomb (Hubbard) repulsion. These are usually referred to as lower and upper Hubbard bands. In our STS experiments, we observe two peaks at V_{LHB} = -77 ± 4 mV and V_{UHB} = 131 ± 4 mV, which we identify as the LHB and UHB, respectively (Fig. 2 of the main manuscript). This yields a measurement of U = 208 ± 4 meV for the effective Hubbard repulsion of the localized Wannier orbital at the Star-of-David center, which gives rise to the flat band. This identification is mainly based on the following arguments. First, the experimental U found here for 1T-$TaSe_2$ is in good agreement with those predicted for similar 1T-TMD monolayers. In particular, previous *ab initio* calculations estimate effective Hubbard U values of 0.3 eV (ref.1) and 0.33 eV (ref.2) in 1T-$NbSe_2$ and 1T-TaS_2, respectively. Furthermore, another piece of evidence is provided by the spatial extension associated with these two peaks. Supplementary Figure 3 shows two conductance maps taken at the maxima of the peaks, i.e. -80 mV and +130 mV for the LHB and UHB, respectively. In both cases the intensity is well localized around the Star-of-David cluster and has its maxima at its center, coincident with the expected spatial localization of the flat band in the CDW state. Lastly, the identical spatial extension associated with these two peaks suggests a common orbital origin.

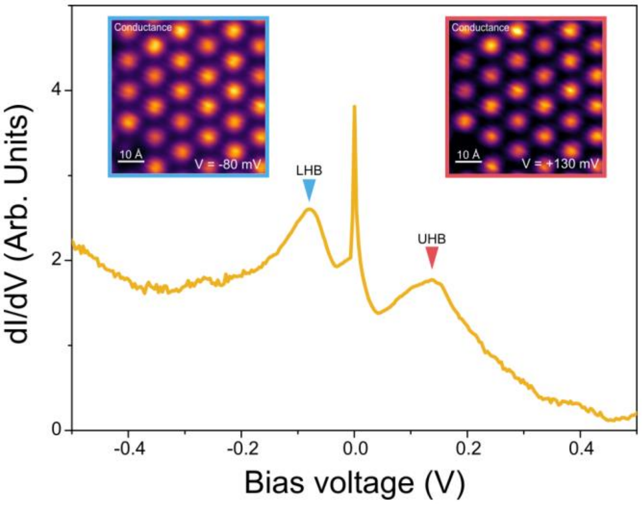

Supplementary Figure 3. Spatial extension of the LHB and UHB. Typical dI/dV curve acquired on the 1T/1H-$TaSe_2$ heterobilayer where the energy location of the LHB and UHB is indicated (T = 0.34 K). The two insets show two conductance maps acquired at the LHB and UHB energy maxima (Conductance maps: $V_{a.c.}$ = 40 μV, B = 0 T, T = 0.34 K).

4. The 1H-$TaSe_2$/BLG substrate

In our experiments, the array of effective magnetic moments in the 1T-$TaSe_2$ monolayer rests on a single layer of 1H-$TaSe_2$, a metal with structural and electronic properties that are reviewed in this section. The H polytype of $TaSe_2$ in the single-layer form is a metal with CDW order[3] below 130 K. At 4.2 K, the CDW develops a commensurate 3 x 3 periodicity that can be easily visualized in STM images (Supplementary Figure 4a). STS measurements show a metallic character of this 2D material although with a partial gap feature of width ≈10 mV centered around E_F, as seen in the dI/dV spectra shown in Supplementary Figures 4b,c. This gap feature has been previously observed in 1H-$TaSe_2$ on two different substrates, BLG and 1T-$TaSe_2$, and identified as the CDW gap[3] and, more recently, as a heavy-fermion gap[4].

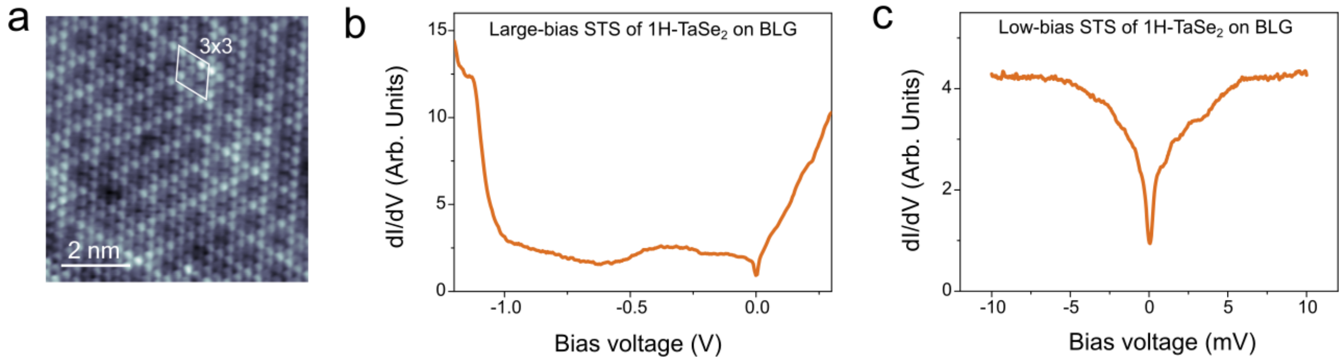

Supplementary Figure 4. Electronic structure of the 1H-$TaSe_2$ substrate. **a**, Atomically resolved STM image of single-layer 1H-$TaSe_2$ with the 3x3 CDW superlattice indicated. Large-bias (**b**) and low-bias (**c**) dI/dV spectra taken on 1H-$TaSe_2$ on BLG at T = 0.34 K.

5. Uncertainty band of Δ

The deviation from the linear behavior of the separation Δ between the two peaks found around E_F was found to be $\overline{\Delta} = 0.15$ mV, as shown in the main manuscript (fig.5e). This is a relatively small energy difference that, however, is larger than the uncertainty threshold imposed by our experimental noise level. In this section, we describe the procedure followed to define this confidence threshold for each *Bz*-dependent series of dI/dV spectra.

In order to estimate the confidence threshold and Δ for a given set of dI/dV spectra, we first realized a linear fit to the Δ values for B ≥ 1T, a magnetic field regime where the Zeeman term ($\Delta \propto g \cdot \mu_B \cdot B_z$) clearly dominates. In most of the cases, the correlation coefficient *r* was found to be $r > 0.99$, which indicates the strict linearity and low uncertainty of Δ with the B field. Supplementary Figure 5 shows one example among the analyzed Δ vs. B_z plots. The uncertainty band was then defined as the region bound by two lines parallel to the fit line and pinned at the two experimental values showing the largest deviation above and below it for B ≥ 1T (green arrows in the example of Supplementary Figure 5). This uncertainty band and the linear fit are then extrapolated down to B = 0 T to quantify the deviations. The deviation from the linear behavior S is defined as $S = \Delta_{exp}(B = 0T) - \Delta_{fit}(B = 0T)$. The upper/lower boundaries of the band mark the uncertainty threshold of the measurements. Deviations smaller than the threshold were not included in the statistical analysis.

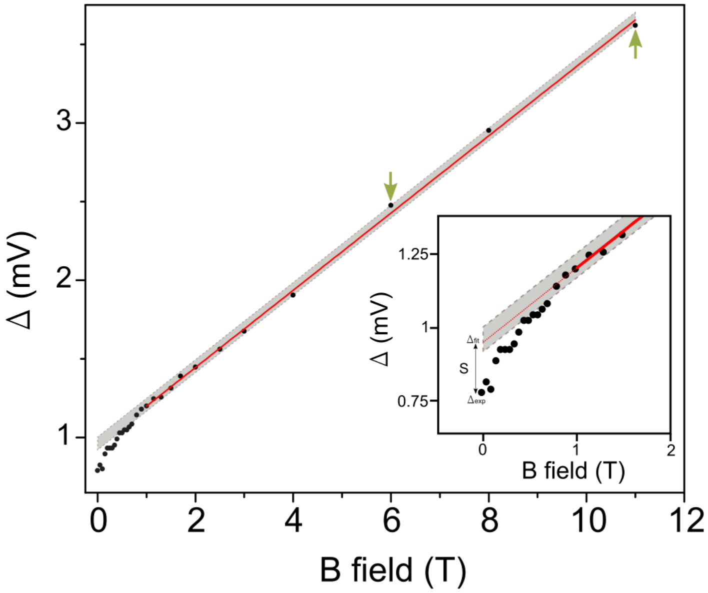

Supplementary Figure 5. Uncertainty band and non-linear behavior of Δ. Example of the analysis of the uncertainty band (grey band) from the linear fit (red line) of a set of values. The inset shows a zoom-in of the main plot in the non-linear region.

6. Measurement of the evolution of Δ with the magnetic field

In order to carry out the statistical analysis of the behavior of Δ with B_z, we measured two different samples, ten different locations (heterobilayers) and, in total, in 36 Star-of-David clusters. The B-dependent STS measurements were all acquired in bulk regions (~10 nm off the edges) of the 1T/1H-heterobilayer and as far as possible from defects previously identified by STM imaging at different bias voltages. At each location, the usual procedure was to measure simultaneously the B-dependence in several (2-4) Star-of-David clusters. These simultaneous STS measurements enabled to rule out instrumental artifacts related to non-linear output values at low magnetic fields as we measured distinct evolutions of Δ. Supplementary Figures 6a,b show an example of these simultaneous B-dependence STS measurements on two neighboring clusters in the range 0 T ≤ B_z ≤ 11 T (only shown up to 2 T) (Supplementary Fig. 6c). As seen, Δ deviates from the linear trend at low fields in both cases. However, in one case the experimental Δ evolves to larger values than Δ_{fit} (negative S) as B_z decreases while in the other case the opposite trend is observed. Our statistical analysis confirms that both trends are frequently seen although with different weight, as shown in the histogram of the occurrence of S (same as that shown in fig. 5e) taking into account its sign.

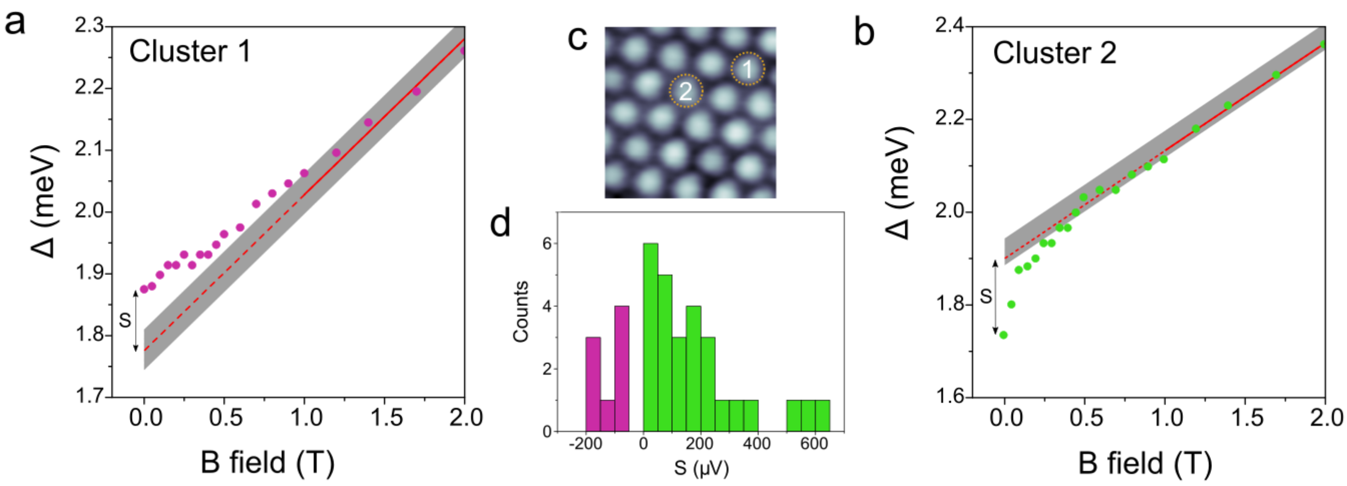

Supplementary Figure 6. Measurements of Δ. a, **b**, Plots of Δ as a function of B_z for two series of dI/dV spectra taken consecutively on the Star-of-David clusters labeled as 1 and 2 in the STM image of **c**. **d**, Histogram of the occurrence of S. The uncertainty band is shown in grey.

7. Absence of magnetism in single-layer 1H-$TaSe_2$

As mentioned in the main manuscript, the emergence of two peaks around E_F admits two plausible interpretations, namely the coherence of the 2D Kondo lattice but also the existence of isolated Kondo impurities (lack of coherence) coupled to a magnetic substrate. The latter would also show a similar zero field splitting as observed on single spins on magnetic substrates[5,6]. To rule out this scenario, we have measured the Kondo fingerprint in the DOS of isolated CoPC molecules (spin ½ in the gas phase) on a monolayer of H-$TaSe_2$. If H-$TaSe_2$ is indeed magnetic, the exchange coupling should split the Kondo peak as observed in the mentioned experiments.

Isolated CoPC molecules (Sigma-Aldrich) were deposited on a single-layer $TaSe_2$ sample at room temperature in a UHV chamber connected to the STM system. The sample was subsequently transferred to the STM carry out the measurements. Supplementary Figure 7 summarizes our main results. The adsorption of individual CoPC molecules on single-layer $TaSe_2$ leads to two different molecular configurations with respect to the substrate (Supplementary Figure 8a). These two types of molecules show a markedly distinct low-lying electronic structure. In one case, the molecules show clear signatures of inelastic excitations at ± 25 meV (Supplementary Fig. 8b), which have been previously identified as the result of S = 0 to S = 1 transition[7–9]. More relevant for our purpose is the case of the type II molecules, which exhibit a sharp Kondo resonance at E_F (Supplementary Figure 7c), in analogy with the Kondo peak we observe in the T/H phase at 4.2 K (fig. 2b in the manuscript). Unlike in the Kondo resonance in the T/H heterostructure, the Kondo peak measured on the molecule does not split around zero bias as the temperature lowers down to 340 mK (Supplementary Figures 7d,e). This result was reproduced in tens of individual type-II molecules using a.c. modulations as low as 50 μeV and using a high density of sampling points (> 20 points/mV). The absence of a splitting clearly rules out the existence of magnetism in the H-$TaSe_2$ substrate. This result is in good agreement with previous XAS/XMCD measurements on monolayers of $TaSe_2$, which report the absence of magnetization in the layer[10]. Lastly, the peak split under magnetic fields as expected for a Kondo resonance.

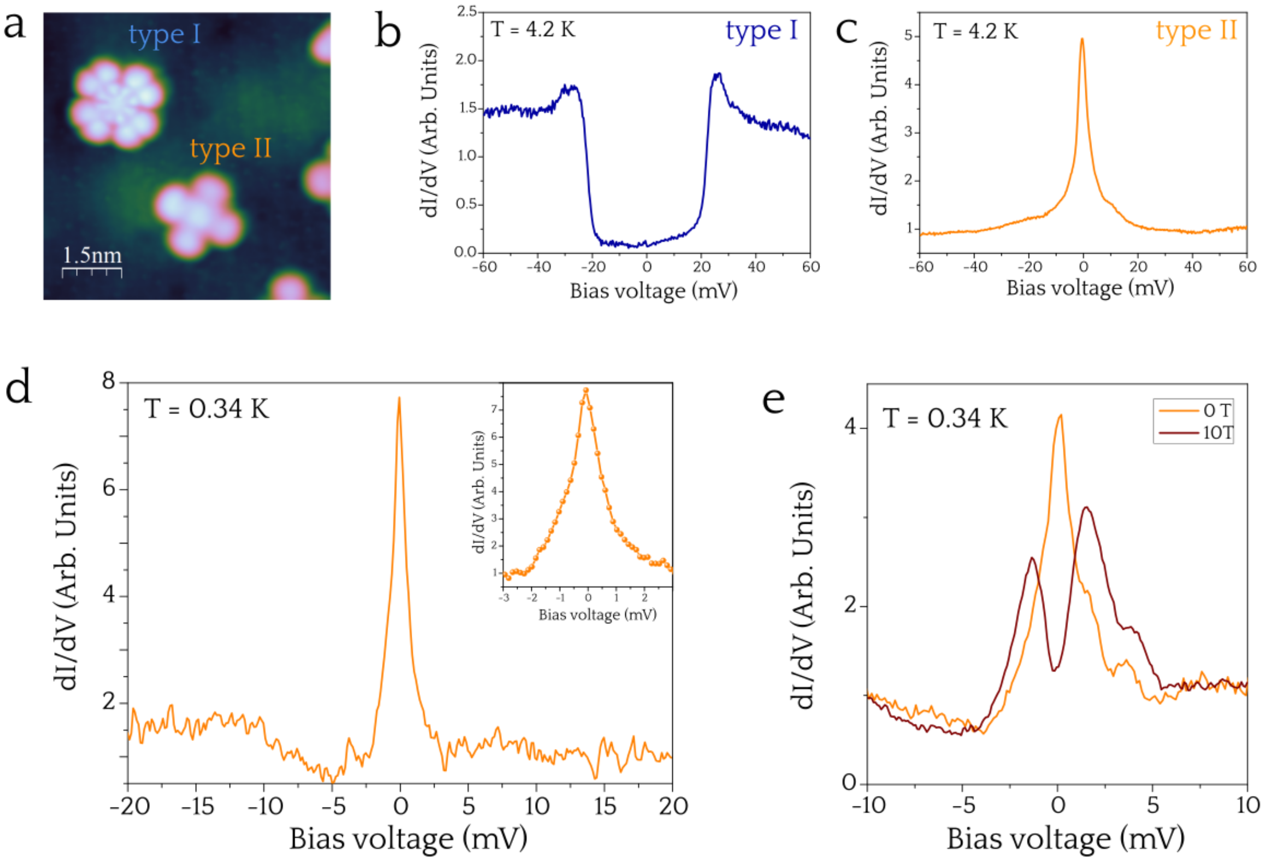

Supplementary Figure 7. Probing magnetism in the 1H-TaSe$_2$ substrate. a, STM topography showing the two configurations of individual CoPC molecules on 1H-TaSe$_2$. **b** and **c**, typical dI/dV spectra for both types of molecules at T = 4.2 K. **d**, dI/dV spectrum on a type-II molecule at T = 0.34 K showing no split around E_F ($V_{a.c.}$ = 50 µV). **e**, Splitting of the Kondo peak due to the Zeeman effect measured at 10 T ($V_{a.c.}$ = 50 µV).

8. Band structure calculations and estimate of Kondo hybridization

The band structure for the H polytype in its original unit cell is shown in Supplementary Figure 8b, showing the half-filled band at the Fermi level, with bandwidth W = 1.2 eV. Supplementary Figure 8b also shows the computed density of states, with a value at E_F of $\rho = 2.5$ eV^{-1}. We have fitted this band structure with a single orbital tight binding model with hoppings up to the 10th neighbor. We then refined the fit by computing the band structure for the 1H phase in the $\sqrt{13} \times \sqrt{13}$ supercell, so that now 13 bands are fitted. This contains more information than just fitting the high symmetry paths of a single band. The final values of the hoppings in eV are given as t_i = (0.087, 0.215, -0.028, -0.006, -0.027, 0.003, -0.006, -0.013, -0.006, 0.010), with a dominating 2nd neighbor hopping[11], and the resulting fitted band is shown in Supplementary Figure 8b.

Next we computed the bands for the 1T polytype in the CDW state, shown in Supplementary Figure 8c. We find the well-known half-filled flat band, of width 25 meV, located inside the large CDW gap of 0.55 eV between the highest fully occupied band and the lowest empty one, in line with previous calculations of 1T-TMDs[12–14]. The flat band is found 40 meV above the highest occupied band. The exact position of the flat band within the CDW gap is known to depend on the functional used[1], but most calculations for 1T-$TaSe_2$ agree on the flat band being isolated from other bands, as we find.

To compute the bands of the 1T/1H system, we assume a perfect lattice match of the two structures in the AA stacking[15], with an interlayer distance of 6.6 Å. The resulting band structure is shown in Supplementary Figure 8d. By comparing the bands with those of the uncoupled 1T and folded 1H structures (aligning the Fermi levels of all three calculations), we observe that there is a small mixing of the bands, as we expect due to the presence of interlayer hopping. We also observe a small overall downward shift of the bands coming from the 1H structure, which implies a small charge transfer from the 1T to the 1H structure.

These findings are consistent with previous work. The 1T/1H $TaSe_2$ structure in the absence of the 1T CDW shows both small interlayer hopping and charge transfer from 1T to 1H[15,16]. Charge transfer can be quantified in our calculation by computing the density of states (DOS) of all three structures: 1T, 1H, and 1T/1H. Integrating the DOS for the 1H structure in the $\sqrt{13} \times \sqrt{13}$ supercell yields a total charge of 13.03 electrons per cell, consistent with a half-filled band in the original cell with one electron per cell. Computing the projected DOS in the 1H layer for the 1T/1H structure and integrating up to the Fermi

level, we find 13.35 electrons, which implies a charge transfer of 0.32 electrons per supercell from the 1T to the 1H layer, consistent with a previous estimate for TaS_2 (Ref.16).

Since we are interested in the interlayer hopping strength for the flat band near the Fermi level, we now consider a tight-binding model which contains the previously fitted 1H band structure in the $\sqrt{13} \times \sqrt{13}$ unit cell, coupled to a single completely flat band at E_F with a constant interlayer hopping V (known as the Kondo hybridization). In addition, we allow for an on-site energy δ_H to account for the downward rigid shift as observed *ab-initio*. Direct comparison of the band structure shows the best agreement between the model and the *ab-initio* calculation for $\delta_H = -50 - 60$ meV and V = 15-20 meV. The bands for the highest values $\delta_H = -60$ meV, $V = 20$ meV are shown in Supplementary Figure 8e. The conclusion of this calculation, revealed by the comparison of Supplementary Figure 8d-e is that the Kondo hybridization in this problem cannot reasonably be any larger than 20 meV. Assuming the value of U = 208 meV extracted from the experiments, this leads to an effective Kondo coupling $J_K = 8V^2/U = 15.4$ meV.

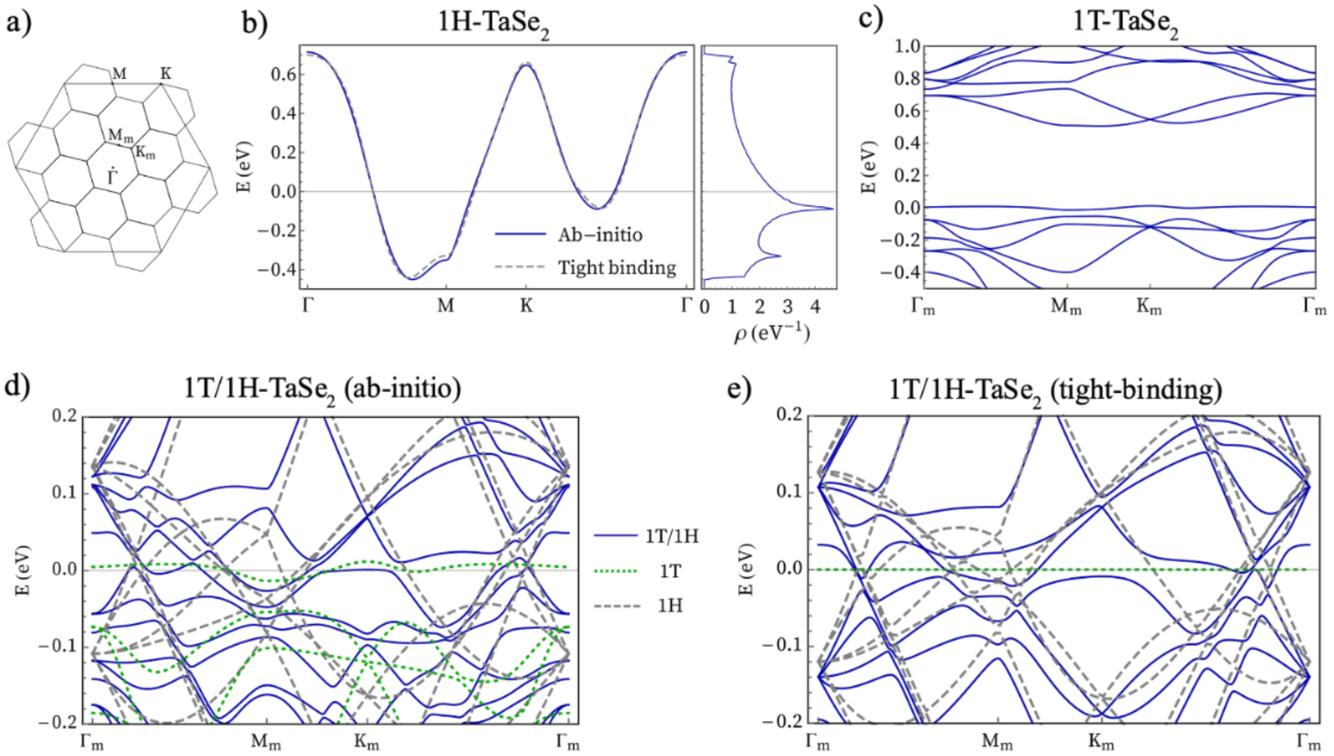

Supplementary Figure 8. Ab-initio band-structure calculations. **a**, Folding scheme showing Brillouin Zones for the 1H and 1T structures in the $\sqrt{13} \times \sqrt{13}$ CDW state. High symmetry points for the latter are labeled with a subscript *m*. **b**, *Ab-initio* band-structure and density of states for single-layer 1H-$TaSe_2$. The tight binding fit is also shown. **c**, *Ab-initio* band-structure of single-layer 1T-$TaSe_2$ in the CDW state. **d**, Close-up of the *ab-initio* band structure of the 1T/1H heterostructure. Bands for the isolated 1H and 1T monolayers are shown as dashed and dotted lines for comparison. **e**, Band structure of the effective tight binding model containing the fitted bands for the 1H layer and a single flat band for the 1T layer, with a Kondo hybridization between them of magnitude V = 20 meV. Isolated bands at V = 0 corresponding to isolated 1H and 1T also shown for comparison.

References

1. Kamil, E. *et al.* Electronic structure of single layer 1T-$NbSe_2$: interplay of lattice distortions, non-local exchange, and Mott–Hubbard correlations. *J. Phys. Condens. Matter* **30**, 325601 (2018).
2. Pasquier, D. & Yazyev, O. V. Ab initio theory of magnetism in two-dimensional 1T-TaS_2. *Phys. Rev. B* **105**, L081106 (2022).
3. Ryu, H. *et al.* Persistent Charge-Density-Wave Order in Single-Layer $TaSe_2$. *Nano Lett.* **18**, 689–694 (2018).
4. Vaňo, V. *et al.* Artificial heavy fermions in a van der Waals heterostructure. *Nature* **599**, 582–586 (2021).
5. Kawahara, S. L. *et al.* Kondo peak splitting on a single adatom coupled to a magnetic cluster. *Phys. Rev. B* **82**, 020406 (2010).
6. Fu, Y.-S., Xue, Q.-K. & Wiesendanger, R. Spin-Resolved Splitting of Kondo Resonances in the Presence of RKKY-Type Coupling. *Phys. Rev. Lett.* **108**, 087203 (2012).
7. Kezilebieke, S., Žitko, R., Dvorak, M., Ojanen, T. & Liljeroth, P. Observation of Coexistence of Yu-Shiba-Rusinov States and Spin-Flip Excitations. *Nano Lett.* **19**, (2019).
8. Wang, Y., Arabi, S., Kern, K. & Ternes, M. Symmetry mediated tunable molecular magnetism on a 2D material. *Commun. Phys.* **4**, (2021).
9. Drost, R., Kezilebieke, S., Lado, J. L. & Liljeroth, P. Real-Space Imaging of Triplon Excitations in Engineered Quantum Magnets. *Phys. Rev. Lett.* **131**, 086701 (2023).
10. Ruan, W. *et al.* Evidence for quantum spin liquid behaviour in single-layer 1T-$TaSe_2$ from scanning tunnelling microscopy. *Nat. Phys.* **17**, (2021).
11. Barnett, R. L., Polkovnikov, A., Demler, E., Yin, W. G. & Ku, W. Coexistence of gapless excitations and commensurate charge-density wave in the 2H transition metal dichalcogenides. *Phys. Rev. Lett.* **96**, 026406 (2006).
12. Zhang, K., Si, C., Lian, C. S., Zhou, J. & Sun, Z. Mottness collapse in monolayer 1T-$TaSe_2$ with persisting charge density wave order. *J. Mater. Chem. C* **8**, 9742–9747 (2020).
13. Jiang, T. *et al.* Two-dimensional charge density waves in TaX_2 (X= S, Se, Te) from first principles. *Phys. Rev. B* **104**, 075147 (2021).
14. Chen, Y. *et al.* Strong correlations and orbital texture in single-layer 1T-$TaSe_2$. *Nat. Phys.* **16**, 218 (2020).
15. Yan, J. A., Dela Cruz, M. A., Cook, B. & Varga, K. Structural, electronic and vibrational properties of few-layer 2H- and 1T-$TaSe_2$. *Sci. Reports 2015 51* **5**, 1–13 (2015).
16. Wang, Z. *et al.* Surface-Limited Superconducting Phase Transition on 1T-TaS_2. *ACS Nano* **12**, 12619–12628 (2018).
